# Association between health care insurance type and rates of visceral surgical procedures in Switzerland. A population-based weighted retrospective cohort study

**DOI:** 10.1186/s12889-026-28164-9

**Published:** 2026-07-03

**Authors:** Kris Rafaisz, Flurina Suter, Sabine Rohrmann, Beat Mueller, Philipp Schuetz, Christian Andreas Nebiker, Alexander Kutz, Tristan Struja

**Affiliations:** 1 Department of Internal Medicine, University Hospital Zurich (USZ), University of Zurich (UZH), Zurich, Switzerland; 2 Medical University Clinic, Kantonsspital Aarau (KSA), Tellstrasse, Aarau, 5001 Switzerland; 3 Division of Chronic Disease Epidemiology, Epidemiology, Biostatistics and Prevention Institute (EBPI), University of Zurich (UZH), Zurich, Switzerland; 4 Medical Faculty of the University of Basel, Basel, Switzerland; 5 Division of Visceral Surgery, Surgery, Kantonsspital Aarau (KSA), Aarau, Switzerland; 6 Massachusetts Institute of Technology, Institute for Medical Engineering and Science, Cambridge, MA USA

**Keywords:** Health care, Insurance, Visceral surgery, Procedure

## Abstract

**Background:**

Indication for visceral surgical procedures should be based on clinical reasoning only and independent of financial incentives. Yet, there is a lack of studies investigating whether insurance type (basic vs. supplementary private) is associated with surgical procedure rates.

**Methods:**

In this study we assessed whether incidence rates in adults with supplementary private insurance undergoing visceral surgical, non-emergency, in-patient procedures from 2012 to 2020 are higher compared to those with basic insurance only in Switzerland. We assessed incidence rates (IR) for basic only and supplementary private insurance stratified over time and by different age groups, and we fitted negative binomial regression models adjusted by inverse probability weights for specific visceral surgical procedures.

We used primary or secondary discharge procedure codes for one of the following procedures: cholecystectomy, fundoplication, sigmoidectomy, rectopexy, haemorrhoidectomy, inguinal, femoral, and umbilical hernia repair.

**Results:**

Of 1,954,119 surgical admissions (median age 63, 53.3% male, 15.3% non-Swiss nationality), 70.5% had basic insurance only. People with supplementary private insurance had a 7% higher probability (IRR, 1.07, 99% CI 1.06–1.07) to have a visceral surgical procedure done compared to people with basic insurance only—a result consistent across different types of procedures including cholecystectomy (IRR, 1.06, 99% CI 1.06–1.06), fundoplication (IRR, 1.09, 99% CI 1.09–1.10), sigmoidectomy (IRR, 1.10, 99% CI 1.10–1.10), rectopexy (IRR, 1.05, 99% CI 1.04–1.06), haemorrhoidectomy (IRR, 1.04, 99% CI 1.03–1.04), and hernia repair (IRR, 1.07, 99% CI 1.07–1.07). Sensitivity analyses, including side procedures, stratification by length of stay, and propensity score matching, suggested robustness of the results.

**Conclusion:**

In this cohort study, supplementary private insurance was independently associated with a higher probability of undergoing a visceral surgical procedure. The role of financial incentives in surgical procedures is still unclear.

**Supplementary Information:**

The online version contains supplementary material available at https://doi.org/10.1186/s12889-026-28164-9.

## Introduction

Since the 1990 s, health care spending generally outpaced economic growth in European countries. In 2020, European Union (EU) countries allocated an unprecedented 10.9% of their gross domestic product (GDP) to health care [1].Switzerland’s health care expenditure in 2020 was 11.8% of its GDP, placing Switzerland at the top in Europe [1].

A defining feature of the Swiss health care system is universal basic health insurance covering a fee-for-service model in outpatient care and case-based payments for in-patients according to the “Swiss Diagnosis Related Groups” (SwissDRG) system [2, 3]. In addition to the universal basic insurance, residents can choose to obtain a supplementary private insurance, granting them additional benefits on top of the basic coverage [3]. These benefits include the ability to choose a physician in hospitals, two-bed rooms or single-bed rooms during hospitalisation, treatment in private clinics, and additional amenities (Supplementary eFigure 1) [3, 4]. Whereas insurers have to accept every application for universal health coverage, applicants for supplementary private insurance need to fill out a health status questionnaire. In case of prior comorbidities, insurers are allowed to reject applicants for supplementary private insurance without explanation [3]. This presumably leads to healthier individuals being more likely to obtain a supplementary private insurance. It is widely speculated whether or not such a selection bias contributes to inefficient health care allocation. From basic insurance, providers receive a specific payment by SwissDRG, which is negotiated nationally on an annual basis [4]. Apart from this, separate contracts exist between providers and private health insurance plans covering benefits and services not included in SwissDRG [4]. In the past, physicians in public hospitals received sizable royalties for treating patients with supplementary private insurance in Switzerland. Even though most public hospitals have abolished revenue sharing, physicians may still experience pressure to increase revenues and reduce costs. Further, many providers in private clinics still receive royalties on a fee-per-performance basis. Current evidence suggests that patients with supplementary private insurance seem to be overtreated, especially in the field of cardiological interventions [5, 6] and orthopaedic surgery [7–10].

The aim of this study was to analyse and assess differences in incidence rates of elective, and thus non-emergency visceral surgical procedures in Switzerland in regard to health insurance plans.

## Methods

The fully-anonymized claims data was provided by the Swiss Federal Statistical Office (FSO, Neuchâtel, Switzerland). In this nationwide cohort study, we included all hospitalisations for adults undergoing visceral surgical procedures as outlined below from January 1 st 2012 until December 31 st 2020.

All hospitals in Switzerland are required to report admissions to the FSO using a unique identifier. To ensure anonymization, the FSO processes the data and provides readmission information in a monthly resolution while assigning a new identifier every time a dataset is released. Importantly, there is no data on procedures performed in an out-patient setting available on a federal level in Switzerland, as this information collection is not mandate by current legislation. The database contains not only information on demographic characteristics such as age, sex, and nationality of the surgical in-patients, the hospital type during stay (i.e., primary university hospital, secondary, and tertiary hospitals), length of stay, and comorbidities (e.g., obesity, diabetes mellitus, coronary artery disease), but also information on the individual diagnoses and surgical procedures performed. Patients were categorized into two groups: patients with basic insurance and patients with supplementary insurance including semi-private and private insurance in one group (Supplementary eFigure 1). Two classifications were used to code the surgical procedures and medical diagnoses: The Swiss Classification of Operations (CHOP) was used to encode the surgical procedures [11], and the International Classification of Diseases, Version 10, German Modification (ICD-10 GM) was used for all medical diagnoses [12]. We defined the visceral surgeries as main or any visceral surgery. Main visceral procedures were defined as the first and most relevant procedure during an admission. Because hospital billing practices may relegate procedures to other positions due to financial reasons, we also included visceral procedures at any position in the dataset. The institutional review board approval, which included a waiver of the requirement of the participants’ informed consent, was provided by the Institutional Review Board of Northwestern Switzerland, as the data was de-identified (EKNZ BASEC Req-2021–01397). This study was conducted in accordance with the principles stated in the Declaration of Helsinki, and it followed the guidelines of Strengthening the Reporting of Observational Studies in Epidemiology (STROBE) [13].

### Eligibility criteria

We excluded pregnant patients, patients younger than 18 years old, patients with a non-elective stay, patients staying in a specialised hospital (according to FSO General Classification of Economic Activities (NOGA) category 85.11B including psychiatric clinics, rehabilitation centers, and other specialized clinics belonging to the fields of gynecology, pediatrics, and geriatrics), patients having a length of stay (LOS) of less than 1 day or more than 28 days, and patients without information on insurance status, or where the payer was not the health insurance itself. We excluded admissions of < 1 day because they primarily represent transfers to other hospitals leading to a heterogenous sample. We excluded patients with LOS > 28 days to ensure that exceptionally severe or atypical cases with prolonged complications would not unduly influence the analysis. We were also concerned about potential unmeasured confounding, as healthcare providers may have been aware of a patient’s clinical condition prior to initiating an intervention. Consequently, treatment decisions may have been driven more by the perceived severity of illness than by the presumed insurance status. This exclusion affected only a small proportion of the cohort (26,908 out of 4,497,304 cases, or 0.6%). To further validate this assumption, we performed a sensitivity analysis further restricting LOS to < 7 days.

### Outcomes

Discharge procedure codes (i.e., CHOP codes) for the visceral surgical procedures were used to identify outcomes. The visceral surgical procedures analysed were cholecystectomy (CHE), fundoplication (FundoP), sigmoidectomy (SigmoidE), rectopexy (RPX), haemorrhoidectomy (HRE), inguinal hernia repair (IHR), femoral hernia repair (FHR), and umbilical hernia repair (UHR). Regarding the CHOP codes for sigmoidectomy, we restricted the procedures to those with a concurrent ICD-10 diagnosis of diverticulosis (K57.30) or diverticulitis (K57.32) of the colon without perforation, abscess, or bleeding (Supplementary eTable 3).

The primary outcome measure was the incidence rate (IR) of main visceral surgical procedures at the primary position. The secondary outcome measure consisted of the IR of main and any visceral surgical procedures combined. In addition, secondary in-hospital safety outcomes included intensive care unit (ICU) stay, ICU ventilation duration, death during hospitalisation, 30-day all-cause readmission, surgical site infection (SSI), haemorrhage, sepsis, thromboembolic event, pneumonia, urinary tract infection (UTI), and major adverse cardiovascular events (MACE) including myocardial infarction, cardiac arrest, and stroke.

### Statistical analysis

We calculated summary statistics for pertinent baseline demographics and major comorbidities. Statistical significance was set at an alpha level of 1% instead of the widely used 5% in order to correct for multiple testing. All significance tests were two-sided. All analyses were done in Stata 17 (Statacorp, USA).

At first, a propensity score (PS) was calculated by logistic regression using insurance status as outcome. Matching variables included sex, age, nationality (Swiss vs. Non-Swiss), year of admission, residency before admission, type of hospital and hospital size, anonymous hospital identifier, the Elixhauser comorbidity score [13], and presence of comorbidities (Supplementary eTable 1). We excluded 2% of the most extreme PS to prevent extreme weights for the inverse-probability weighted (IPW) analysis. For covariate balance after IPW, a standardized mean difference (SMD) less than 0.10 was considered appropriate [14]. IR and IR ratios (IRR) were calculated by a negative binomial regression model with dispersion at the outcomes’ means, robust standard errors, and adjustment by IPW according to the propensity score.

Several sensitivity analyses were performed to assess the robustness of our results; specifically, we calculated IR and IRR in crude, negative binomial regression models first. Second, we limited the IPW model to patients with a LOS of 1 to 7 days, taking into consideration potential procedure-related complications leading to prolonged hospitalisation time. Third, a 1:1 PS-matching was performed using the nearest neighbour method with a maximum calliper of 0.01 on the propensity score scale.

### Person-time at risk

Although individual level data were available, this only included data on in-hospital stays. However, we did not have information on the individual person-time necessary for our negative binomial regression model. As a work-around, we used publicly available Swiss census data from the FSO at the end of each study year stratified by age and sex [15]. We accumulated procedure counts on distinct strata per admission year, age, and sex. Specifically, we counted the number of procedures for every distinct stratum paired with the published number of persons in the same stratum, correspondingly. As an example, there were 39,204 male residents at the age of 70, among whom 23 sigmoidectomy procedures were recorded in 2019. Ignoring deceased or migrating people, we assumed one person-year for every resident. IR curves were plotted after accumulating our dataset according to either age in deciles or monthly bins; additionally, we fitted a kernel-weighted local polynomial smoothing illustrating changes over time. The difference of annual treatments in Switzerland was calculated based on the IR difference presuming a population of 6.88 million (i.e., average population from 2012 to 2020 in Switzerland, ≥ 18 years old) and a supplementary private insurance percentage of 30% [3].

## Results

### Study population

**Fig. 1:**
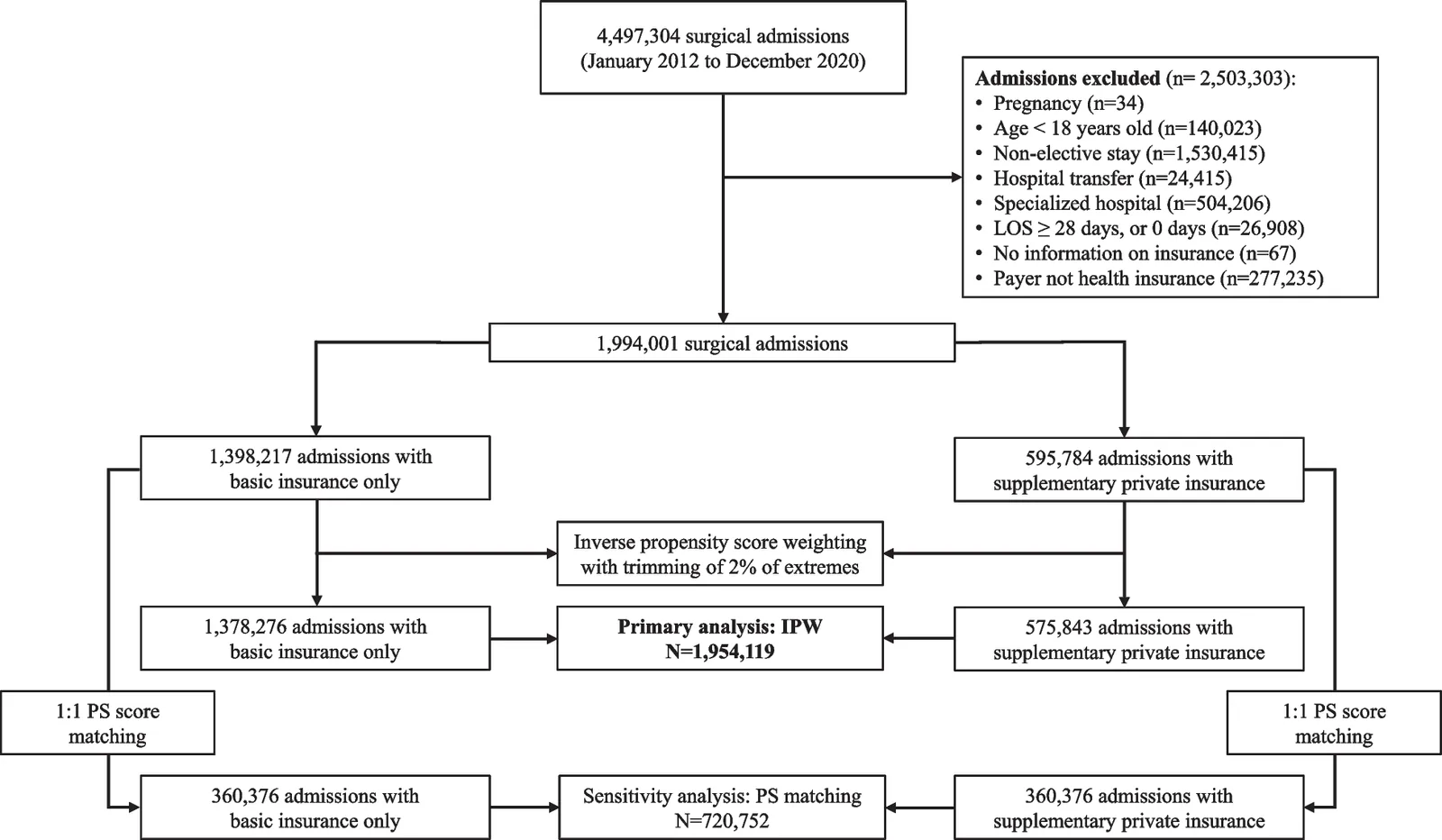
Flow chart of the patient selection process

A total of 1,954,119 surgical admissions (median age 63 years [interquartile range (IQR) 51–73], 53.3% male, 15.3% non-Swiss nationality) were included in the study, of which 70.5% of admissions had basic insurance (Table 1 and Fig. 1). People with basic insurance accounted for 181,238 main visceral surgical procedures, and people with supplementary private insurance accounted for 67,284 main procedures (Supplementary eTable 2). Among hospitalisations with main visceral surgical procedures only, we found 248′522 admissions with a median age of 58 years (IQR 46–69), with male sex in 65.5%, non-Swiss nationality in 18.5%, and basic insurance in 72.9% of the cases (Supplementary eTable 2). Apart from nationality and age, baseline characteristics were well balanced after weighting. The propensity score’s density based on the insurance class showed a good area of common support before and after adjustment (Supplementary eFigure 2 and eFigure 3).

**Table 1:** Baseline characteristics for both patients receiving surgery and those serving as control (for baseline characteristicts of patients with surgery only see supplementary eTable2)

	**Total** ***N* = 1,954,119**	**Basic insurance** ***N* = 1,378,276**	**Supplementary private insurance** ***N* = 575,843**	**SMD after IPW**
Age, years (median, IQR)	63 (51–73)	61 (48–72)	67 (57–75)	0.14
Non-Swiss nationality, n (%)	298,825 (15.3%)	252,281 (18.3%)	46,544 (8.1%)	0.13
Male sex, n (%)	1,041,991 (53.3%)	747,456 (54.2%)	294,535 (51.1%)	0.01
Elixhauser comorbidity score (median, IQR)	1.00 (0.00–2.00)	1.00 (0.00–2.00)	1.00 (0.00–2.00)	0.00
Charlson index (median, IQR)	0.00 (0.00–1.00)	0.00 (0.00–1.00)	0.00 (0.00–1.00)	0.01
Living not at home, n (%)	20,220 (1.0%)	17,474 (1.3%)	2,746 (0.5%)	0.04
*Hospital type, n (%)*				
University	240,008 (12.3%)	196,354 (14.2%)	43,654 (7.6%)	0.07
Tertiary	1,103,649 (56.5%)	817,404 (59.3%)	286,245 (49.7%)	0.02
Secondary, large	249,491 (12.8%)	149,003 (10.8%)	100,488 (17.5%)	0.04
Secondary, medium	286,614 (14.7%)	171,989 (12.5%)	114,625 (19.9%)	0.04
Secondary, small	74,357 (3.8%)	43,526 (3.2%)	30,831 (5.4%)	0.02
*Year, n (%)*				
2012	215,370 (11.0%)	146,591 (10.6%)	68,779 (11.9%)	0.01
2013	220,239 (11.3%)	152,629 (11.1%)	67,610 (11.7%)	0.01
2014	226,922 (11.6%)	157,967 (11.5%)	68,955 (12.0%)	0.01
2015	227,787 (11.7%)	159,456 (11.6%)	68,331 (11.9%)	0.01
2016	226,424 (11.6%)	160,367 (11.6%)	66,057 (11.5%)	0.00
2017	219,181 (11.2%)	156,593 (11.4%)	62,588 (10.9%)	0.01
2018	214,305 (11.0%)	153,066 (11.1%)	61,239 (10.6%)	0.01
2019	209,386 (10.7%)	150,877 (10.9%)	58,509 (10.2%)	0.01
2020	194,505 (10.0%)	140,730 (10.2%)	53,775 (9.3%)	0.01
Length of stay, days (median, IQR)	3 (2–6)	3 (2–6)	3 (2–7)	0.01
*Obesity, n (%)*				
BMI < 30.0 kg/m^2^	1,892,620 (96.9%)	1,325,309 (96.2%)	567,311 (98.5%)	0.06
BMI 30.0–34.9 kg/m^2^	17,591 (0.9%)	13,498 (1.0%)	4,093 (0.7%)	0.00
BMI 35.0–39.9 kg/m^2^	21,682 (1.1%)	18,828 (1.4%)	2,854 (0.5%)	0.04
BMI ≥ 40.0 kg/m^2^	22,226 (1.1%)	20,641 (1.5%)	1,585 (0.3%)	0.06
Comorbidities, n (%)				
Dyslipidaemia	191,902 (9.8%)	131,604 (9.5%)	60,298 (10.5%)	0.02
OSA	36,159 (1.9%)	27,313 (2.0%)	8,846 (1.5%)	0.01
Diabetes Mellitus	171,357 (8.8%)	129,802 (9.4%)	41,555 (7.2%)	0.01
Hypertension	575,129 (29.4%)	398,536 (28.9%)	176,593 (30.7%)	0.03
Coronary artery disease	153,619 (7.9%)	103,900 (7.5%)	49,719 (8.6%)	0.02
Atrial fibrillation	91,642 (4.7%)	61,545 (4.5%)	30,097 (5.2%)	0.02
Peripheral artery disease	56,558 (2.9%)	41,687 (3.0%)	14,871 (2.6%)	0.00
Cerebrovascular disease	27,656 (1.4%)	20,136 (1.5%)	7,520 (1.3%)	0.00
Chronic kidney disease	73,913 (3.8%)	53,889 (3.9%)	20,024 (3.5%)	0.00
End-stage kidney disease	11,539 (0.6%)	9,249 (0.7%)	2,290 (0.4%)	0.01
COPD	43,080 (2.2%)	32,794 (2.4%)	10,286 (1.8%)	0.00
Solid malignancy	258,128 (13.2%)	174,915 (12.7%)	83,213 (14.5%)	0.02
Hematologic malignancy	8,907 (0.5%)	5,810 (0.4%)	3,097 (0.5%)	0.00
Alcohol abuse	12,766 (0.7%)	10,991 (0.8%)	1,775 (0.3%)	0.03
Liver cirrhosis	5,615 (0.3%)	4,705 (0.3%)	910 (0.2%)	0.01
Congestive heart disease	12,502 (0.6%)	9,328 (0.7%)	3,174 (0.6%)	0.00

*Abbr.*: *SMD* Standardized mean difference, *IPW* Inverse probability weighting, *IQR* Interquartile range, *BMI *Body mass index, *OSA* Obstructive sleep apnoea, *COPD* Chronic obstructive pulmonary disease, *N/A *Not applicable

### Trends of visceral surgical procedures

**Fig. 2:**
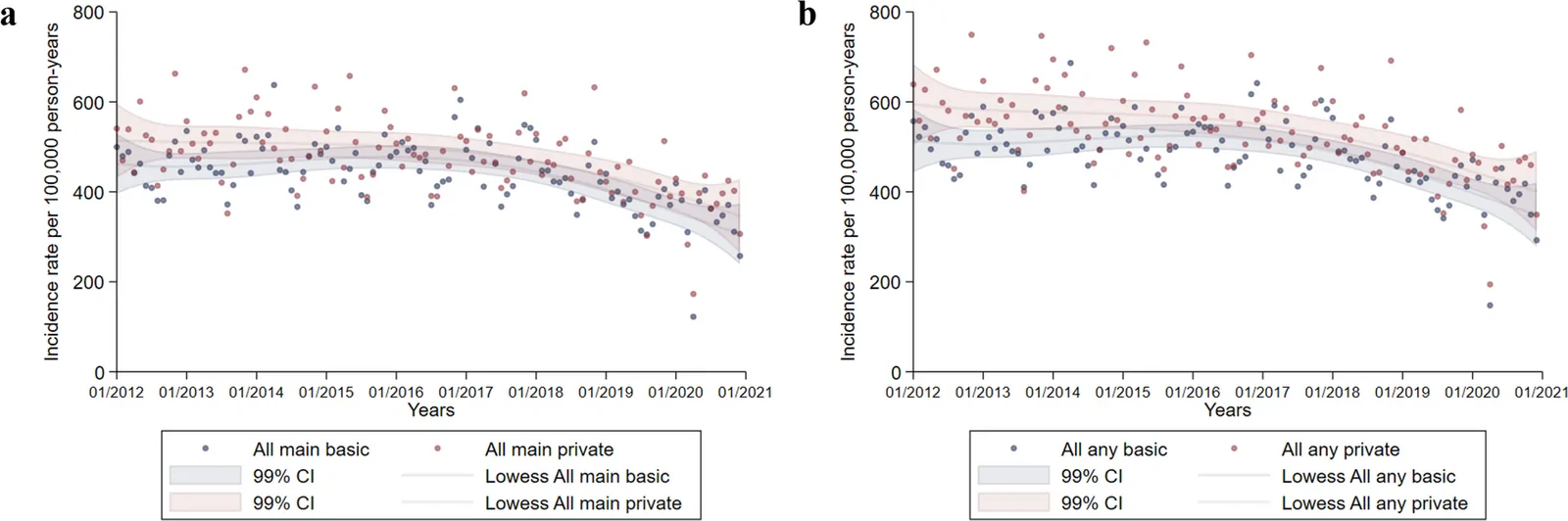
Incidence rates for main visceral surgical procedures only (**a**) and any visceral surgical procedures (**b**) over study years, according to health insurance status. Abbr.: 99% CI, confidence interval

Across the study period, we observed a stable rate of visceral surgical procedures for both people with basic insurance only and those with supplementary private insurance. However, an overall decreasing trend started in 2017 which can be explained by new regulations limiting inguinal hernia repairs and haemorrhoidectomy procedures to be primarily performed in an outpatient setting (Fig. 2A for main procedures, and Fig. 2B for any procedures, respectively) [16]. Due to Swiss legislation, data on procedures performed in an outpatient setting are not gathered in a federal dataset. Cholecystectomy, haemorrhoidectomy, and inguinal hernia repairs individually showed a downward trend over the years; fundoplication, sigmoidectomy, rectopexy, and umbilical hernia repairs showed an upward trend first and a drop over time, especially in 2020 during the COVID-19 pandemic. Femoral hernia repairs were stable over the years (Supplementary eFigure 4). Up to the age of 80 years there was a trend towards higher procedure rates, however, the group above 89 years showed a decline in procedure rates, albeit with wide confidence intervals (CI) (Supplementary eFigure 5). There was a distinction on IRs when comparing basic and supplementary private insured people over the age groups. Below the age of 60 years, people with basic insurance had higher IRs; however, above the age of 60 years, we observed higher IRs among people with supplementary private insurance.

### Outcome data and adverse events

We assessed a selection of the most common adverse outcomes as substitute for quality of care among patients undergoing a visceral surgical procedure (Table 2). No apparent and clinically meaningful differences were observed between the two insurance classes regarding in-hospital death (basic insurance only: 0.1% vs. supplementary private insurance: 0.1%) and 30-day all-cause readmission (basic insurance only: 2.0% vs. supplementary private insurance: 2.2%). Similar findings were observed for intensive care unit (ICU) stay duration, ICU ventilation duration, in-hospital death, and selected adverse outcomes such as surgical site infection (SSI), haemorrhage, major adverse cardiovascular event (MACE), sepsis, thromboembolic event, pneumonia, and urinary tract infection (UTI).

**Table 2:** Subset of the total study cohort with in-hospital adverse outcomes only

Outcome	Insurance	Total	Cholecystectomy	Fundoplication	Sigmoidectomy	Rectopexy	Haemorrhoid-ectomy	Hernia repairs
	**Both**	***N* = 248,522**	***N* = 67,140**	***N* = 3,996**	***N* = 13,792**	***N* = 3,279**	***N* = 18,197**	***N* = 142,118**
	**Basic**	***N* = 181,238**	***N* = 51,602**	***N* = 2,802**	***N* = 9,434**	***N* = 2,144**	***N* = 12,738**	***N* = 102,518**
	**Private**	***N* = 67,284**	***N* = 15,538**	***N* = 1,194**	***N* = 4,358**	***N* = 1,135**	***N* = 5,459**	***N* = 39,600**
ICU stay, n (%)	Both	4,344 (1.7%)	961 (1.4%)	355 (8.9%)	2,042 (14.8%)	174 (5.3%)	53 (0.3%)	759 (0.5%)
	Basic	3,116 (1.7%)	735 (1.4%)	228 (8.1%)	1,441 (15.3%)	123 (5.7%)	35 (0.3%)	554 (0.5%)
	Private	1,228 (1.8%)	226 (1.5%)	127 (10.6%)	601 (13.8%)	51 (4.5%)	18 (0.3%)	205 (0.5%)
ICU stay duration if admitted to ICU, hours	Both	22 (18 to 39)	22 (17 to 32)	22 (18 to 29)	23 (19 to 45)	21 (17 to 25)	20 (14 to 29)	21 (15 to 26)
	Basic	22 (18 to 40)	22 (17 to 32)	22 (18 to 27)	23 (19 to 45)	21 (18 to 26)	20 (14 to 31)	21 (16 to 27)
	Private	22 (18 to 33)	21 (16 to 29)	23 (18 to 37)	23 (20 to 46)	21 (17 to 25)	21 (12 to 29)	21 (12 to 24)
ICU ventilation, n (%)	Both	734 (0.3%)	192 (0.3%)	86 (2.2%)	294 (2.1%)	24 (0.7%)	7 (0.0%)	131 (0.1%)
	Basic	558 (0.3%)	156 (0.3%)	52 (1.9%)	214 (2.3%)	20 (0.9%)	5 (0.0%)	111 (0.1%)
	Private	176 (0.3%)	36 (0.2%)	34 (2.8%)	80 (1.8%)	4 (0.4%)	2 (0.0%)	20 (0.1%)
ICU ventilation duration if ventilated, hours	Both	16 (8 to 24)	16 (8 to 32)	16 (8 to 32)	16 (8 to 32)	16 (8 to 24)	12 (8 to 12)	12 (8 to 24)
	Basic	16 (8 to 24)	16 (8 to 32)	13 (8 to 28)	16 (8 to 24)	16 (8 to 24)	12 (8 to 12)	12 (8 to 24)
	Private	16 (8 to 32)	14 (8 to 32)	16 (8 to 32)	16 (8 to 40)	24 (16 to 144)	7.5 (3 to 12)	8 (8 to 23.5)
Death during admission, n (%)	Both	140 (0.1%)	38 (0.1%)	4 (0.1%)	55 (0.4%)	12 (0.4%)	1 (0.0%)	30 (0.0%)
	Basic	94 (0.1%)	21 (0.0%)	2 (0.1%)	36 (0.4%)	7 (0.3%)	1 (0.0%)	27 (0.0%)
	Private	46 (0.1%)	17 (0.1%)	2 (0.2%)	19 (0.4%)	5 (0.4%)	0 (0.0%)	3 (0.0%)
30 day all-cause readmission, n (%)	Both	5,167 (2.1%)	1,632 (2.4%)	117 (2.9%)	483 (3.5%)	121 (3.7%)	366 (2.0%)	2,448 (1.7%)
	Basic	3,711 (2.0%)	1,233 (2.4%)	78 (2.8%)	331 (3.5%)	83 (3.9%)	265 (2.1%)	1,721 (1.7%)
	Private	1,456 (2.2%)	399 (2.6%)	39 (3.3%)	152 (3.5%)	38 (3.3%)	101 (1.9%)	727 (1.8%)
Surgical site infection, n (%)	Both	1,407 (0.6%)	417 (0.6%)	22 (0.6%)	562 (4.1%)	21 (0.6%)	52 (0.3%)	333 (0.2%)
	Basic	1,086 (0.6%)	332 (0.6%)	16 (0.6%)	422 (4.5%)	17 (0.8%)	38 (0.3%)	261 (0.3%)
	Private	321 (0.5%)	85 (0.5%)	6 (0.5%)	140 (3.2%)	4 (0.4%)	14 (0.3%)	72 (0.2%)
Haemorrhage, n (%)	Both	4,898 (2.0%)	950 (1.4%)	78 (2.0%)	876 (6.4%)	89 (2.7%)	434 (2.4%)	2,471 (1.7%)
	Basic	3,635 (2.0%)	765 (1.5%)	54 (1.9%)	607 (6.4%)	63 (2.9%)	336 (2.6%)	1,810 (1.8%)
	Private	1,263 (1.9%)	185 (1.2%)	24 (2.0%)	269 (6.2%)	26 (2.3%)	98 (1.8%)	661 (1.7%)
MACE, n (%)	Both	1,422 (0.6%)	326 (0.5%)	23 (0.6%)	171 (1.2%)	27 (0.8%)	59 (0.3%)	816 (0.6%)
	Basic	1,012 (0.6%)	244 (0.5%)	22 (0.8%)	121 (1.3%)	19 (0.9%)	41 (0.3%)	565 (0.6%)
	Private	410 (0.6%)	82 (0.5%)	1 (0.1%)	50 (1.1%)	8 (0.7%)	18 (0.3%)	251 (0.6%)
Sepsis, n (%)	Both	309 (0.1%)	121 (0.2%)	13 (0.3%)	125 (0.9%)	7 (0.2%)	5 (0.0%)	38 (0.0%)
	Basic	234 (0.1%)	96 (0.2%)	10 (0.4%)	89 (0.9%)	5 (0.2%)	3 (0.0%)	31 (0.0%)
	Private	75 (0.1%)	25 (0.2%)	3 (0.3%)	36 (0.8%)	2 (0.2%)	2 (0.0%)	7 (0.0%)
Thromboembolic event, n (%)	Both	276 (0.1%)	79 (0.1%)	25 (0.6%)	72 (0.5%)	11 (0.3%)	5 (0.0%)	84 (0.1%)
	Basic	192 (0.1%)	61 (0.1%)	16 (0.6%)	49 (0.5%)	7 (0.3%)	3 (0.0%)	56 (0.1%)
	Private	84 (0.1%)	18 (0.1%)	9 (0.8%)	23 (0.5%)	4 (0.4%)	2 (0.0%)	28 (0.1%)
Pneumonia, n (%)	Both	585 (0.2%)	199 (0.3%)	45 (1.1%)	174 (1.3%)	29 (0.9%)	2 (0.0%)	136 (0.1%)
	Basic	463 (0.3%)	165 (0.3%)	32 (1.1%)	139 (1.5%)	22 (1.0%)	2 (0.0%)	103 (0.1%)
	Private	122 (0.2%)	34 (0.2%)	13 (1.1%)	35 (0.8%)	7 (0.6%)	0 (0.0%)	33 (0.1%)
Urinary tract infection, n (%)	Both	1,259 (0.5%)	286 (0.4%)	42 (1.1%)	479 (3.5%)	127 (3.9%)	51 (0.3%)	274 (0.2%)
	Basic	908 (0.5%)	230 (0.4%)	30 (1.1%)	327 (3.5%)	81 (3.8%)	38 (0.3%)	202 (0.2%)
	Private	351 (0.5%)	56 (0.4%)	12 (1.0%)	152 (3.5%)	46 (4.1%)	13 (0.2%)	72 (0.2%)

Data are presented as median (IQR) for continuous measures, and n (%) for categorical measures

*Abbr.*: *Basic* Basic insurance, Private, supplementary private insurance, Both, basic and supplementary private insurance

### Comparison of incidence rates between the groups

The lowest IPW adjusted IRs were found in main femoral hernia repair; specifically, we observed 6.10 procedures per 100,000 person-years among people with basic insurance, and 6.56 procedures per 100,000 person-years among people with supplementary private insurance. Rectopexy as main procedure had similar results. In contrast, the highest IPW adjusted IRs were found in main inguinal hernia repair, where we observed a total of 306.30 procedures per 100,000 person-years and 329.46 procedures per 100,000 person-years in basically and privately insured people, respectively. Cholecystectomy as a main procedure had the second highest IRs (Supplementary eTable 4). We calculated an absolute number of 793 additional annual main visceral surgical procedures in people with supplementary private insurance. These surplus procedures differed from 8 additional main rectopexy procedures to 479 additional inguinal hernia repairs.

**Fig. 3:**
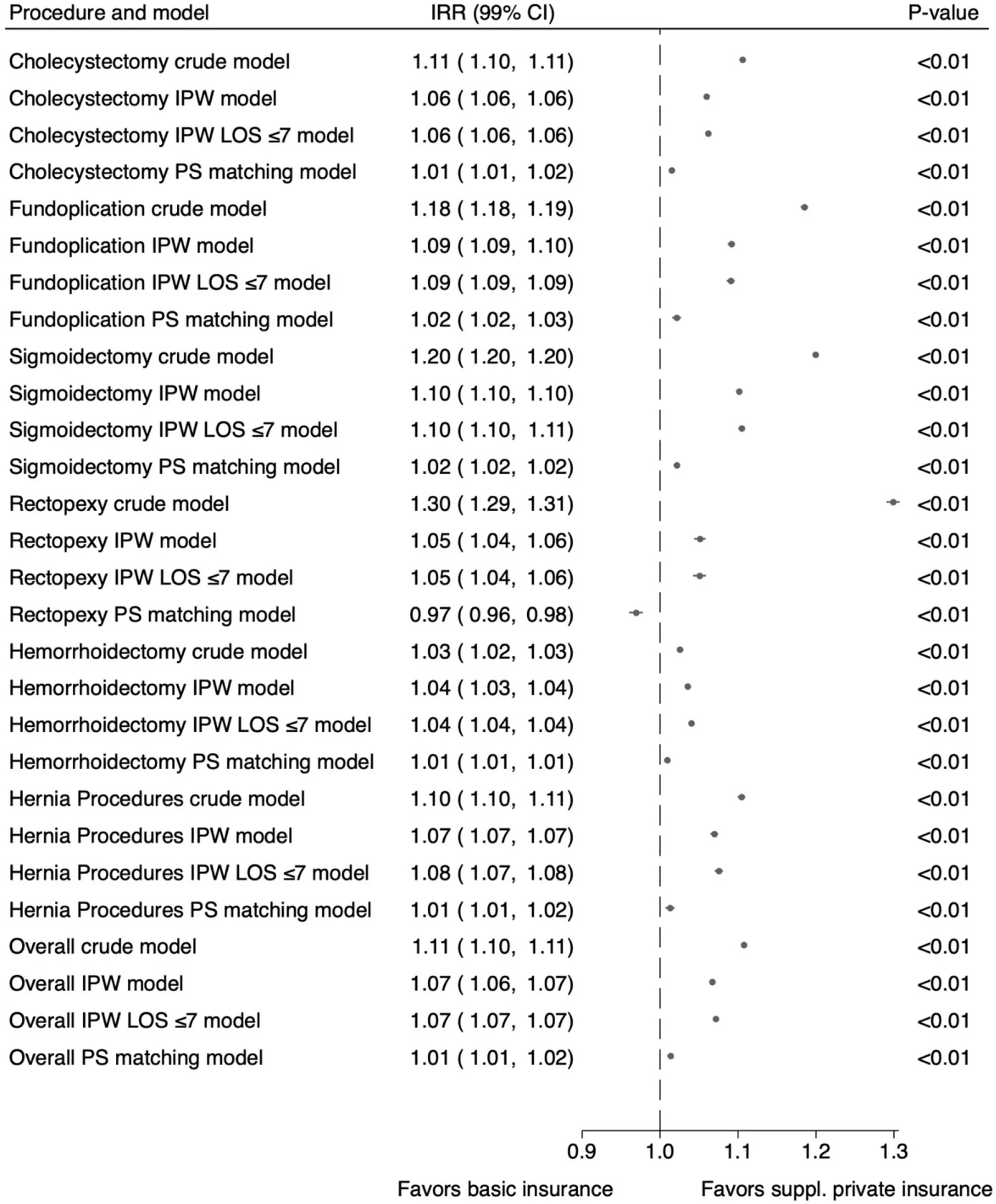
Forest plot of incidence rate ratios for main procedures and models. Note: Inconsistencies with borderline confidence intervals are due to rounding. *P*-value < 0.01 denotes a significant result at the 1% alpha level. Legend: IRR, incidence rate ratio; CI, confidence interval; crude model, model between outcome and insurance class; IPW model, inverse probability weighted model; IPW LOS ≤ 7 model, IPW length of stay ≤ 7 days model; PS matching model, propensity score matching model

In a Forest plot, we depicted IRRs for every main visceral surgical procedure (Fig. 3). When looking at all procedures pooled, we saw an IRR of 1.07 (99% CI 1.06–1.07) in favour of patients with supplementary private insurance. This result was consistent across different types of procedures chosen including cholecystectomy (IRR, 1.06, 99% CI 1.06–1.06), fundoplication (IRR, 1.09, 99% CI 1.09–1.10), rectopexy (IRR, 1.05, 99% CI 1.04–1.06), and hernia repair (IRR, 1.07, 99% CI 1.07–1.07).

These results were also true, when redrawing the Forest plot for any visceral surgical procedure, whereas the IRRs did not change markedly, and the intervention codes were not coded as main procedure. (Supplementary eFigure 5).

### Sensitivity analyses

The IRR results did not change substantially when restricting the cohort to patients with a LOS of 1 to 7 days instead of 1 to 28 days (Supplementary eTable 4 and eTable 5). Focusing on any visceral surgical procedure, the IRs for femoral and umbilical hernia repairs doubled; the remaining results did not change relevantly (Supplementary eTable 5). When using the PS matching approach, there was a trend for a lower absolute rate difference between patients with basic insurance only and those with supplementary private insurance. We also performed a further sensitivity analysis by using coarsened exact matching before the negative binomial regression. The results showed a regression towards the null (see supplementary eTable 4 and eTable 5).

## Discussion

People with supplementary private insurance had a 7% higher chance to be treated by cholecystectomy, fundoplication, sigmoidectomy, rectopexy, haemorrhoidectomy, IHR, FHR, and UHR as opposed to people with basic insurance only translating into 793 additional main visceral surgical procedures in Switzerland every year. Independent of patient characteristics, we found a treatment imbalance, potentially suggesting misallocation of resources. Data from the FSO in Switzerland suggest that people with supplementary private insurance are healthier, have a higher socioeconomic status (i.e., higher education, more disposable income), and seek a physician’s consultation more frequently compared to people with basic insurance only [3]. It is debated whether people who invest more in health insurance also want to optimize their return by seeking more care.

Both IPW and propensity score matching (PSM) have distinct advantages and drawbacks. Each method differs in suitability depending on the scenario presented, e.g., availability of treatment and comparator patients, and number of compared treatments [17]. Furthermore, PSM and inverse probability of treatment weighting (IPTW) might not always result in the same study conclusions; therefore, results should be viewed with consideration of differences in technical methodology, and differences in measurement and interpretation of the treatment effect each method shows [17]. Although all methods available try to model selection bias by making a treatment and control group as similar as possible, no single gold-standard approach on how to correct for selection bias exists [18]. We pre-hoc defined IPW as our primary analysis as we considered that PSM is wasteful of data, estimates the average treatment effect among the treated (ATT) [19, 20], and might introduce bias especially given our nationwide sample. IPW on the other hand is sensitive to outliers adding extreme weights to the analysis, but is better in estimating the average treatment effect (ATE) which is more accurate in our use case [19, 20]. To prevent distortion of the results by outlier, we trimmed cases with 2% of the most extreme weights from our analysis. With this approach, we tried to present a nuanced analysis giving the readers a way to interpret the impact of analytical choices and gauge the size of the potential true effect.

Our findings have also been replicated in other medical specialties in Switzerland. We recently conducted a nationwide study comparing cardiovascular procedure rates in patients with supplementary private and basic insurance only [9]. We found that people with a supplementary private insurance compared to those with basic insurance only had an 11% higher likelihood of receiving non-emergency cardiovascular procedures [6]. In orthopaedics, a study found that supplementary private insurance was associated with a higher rate of hip replacement [10]. Another report observed a higher incidence rate of spinal surgery in patients older than 70 years [8], and Muheim et al. showed an association between arthroscopic partial meniscectomy and supplementary private insurance [9]. Further work found geographical variations in Switzerland in procedure rates for both hip and knee arthroplasty, and cardiologic interventions including implantations of pacemakers, implantable cardioverter defibrillators, and cardiac resynchronization therapy devices suggesting biased indications by physicians [5, 7].

While regional variations exist, there are also large discrepancies in the proportions of in-patient procedures for a given surgery in developed countries [21], even though there is no medical reason for the incidences to vary considerably among similar countries. While over 99% of IHR were performed in an in-patient setting in Germany between 2012 and 2020, in Croatia IHR procedures performed in an in-patient setting fell from 99.9% to 67.1% over the same time frame [22].

Likewise, in-patient cholecystectomy rates remained stable at over 90% in Germany from 2012 to 2020, the rate decreased from 94.8% to 60.8% in France over the same time [22].

In an effort to control health care expenditures, many Swiss cantons enforced an outpatient treatment first policy which is visible in our data by a sharp decline in procedure rates for haemorrhoidectomy and IHR starting in 2017 [16]. This is reflected in data by the Organization for Economic Cooperation and Development (OECD) showing that procedure rates in Switzerland remained stable for cholecystectomy, but declined for the IHR, while the opposite trends were visible in Austria [22]. In 2019, the Federal Office of Public Health of Switzerland extended this rule to the federal level and six common surgical procedures previously conducted in an in-patient setting unless a patient fulfils certain criteria for frailty [23]. For instance, extension of IHR to simultaneous bilateral repair is still billable in the in-patient setting and has favourable reimbursement. This is problematic as an analysis found that unilateral hernia repairs in patients with basic insurance only resulted in a net loss of approx. EUR –844 per case, whereas procedures in patients with supplementary private insurance generated a revenue of approx. EUR + 1,119 per case [24]. However, CHOP codes were merged in 2017—the same year as new regulations took effect [16]—and we were unable to provide any evidence on that matter.

While new regulations in Switzerland enforce a shift from the in-patient setting to the outpatient setting, it is debated whether quality of care can be maintained under these conditions. A Swiss report looked at the recently imposed regulations and showed a decrease in health care costs without an effect on follow-up hospitalisations or downstream outpatient costs [25]. The prospective observational HerStAmb-study from Switzerland compared outpatient IHR to in-patient IHR and could not find any significant differences in re-admission rates, complications, and in quality of life (QoL) [26]. As royalties from supplementary private insurance plans apply to in-patient setting only, providers could be willing to hospitalise patients with supplementary private insurance in pursuit of increased revenue as suggested in case of arthroscopic meniscal surgery in Switzerland [9].

Such potential drivers also exist internationally. A US study from Florida observed in a total of 103,183 cases between 2009 and 2015 that one of the top factors associated with IHR and treatment setting was private insurance coverage followed by income [27]. Furthermore, substantial reimbursement differences exist in the potential surgical approaches for IHR [27]. In such a scenario, it is likely that financial incentives drive the decision for or against a certain procedure type.

Over the last two decades, recommendations on the management of diverticular disease by the European Society of Coloproctology (ESCP) changed substantially [28]. Previously, non-emergency sigmoid resection after the second episode of uncomplicated diverticulitis was widely recommended to prevent further episodes. While sigmoidectomy is effective in minimizing recurrent episodes of diverticulitis, multiple cohort studies have shown that complications are most likely to happen during the first episode and prophylactic surgery to prevent complications from further episodes is not as beneficial as previously anticipated [28].

Clinicians often seek detailed information to accurately assess the risk of an intervention when counselling patients about whether to proceed with a procedure. Using retrospective coding datasets may not always offer the level of granularity required to fully capture such detailed information which is why we tried to include as many relevant complications as possible.

The frequency of adverse events in our cohort was similar to international data. A retrospective analysis of nationwide German data by Baum et al. found an in-hospital mortality of 0.04% (95% CI 0.03% to 0.05%) for inguinal hernia repairs and of 0.40% (95% CI 0.39% to 0.41%) for cholecystectomy [29]. Similar to our findings, a systematic review including more than 150 studies reporting outcomes for more than half a million patients found the following outcomes for perioperative complications: thromboembolic events 0.13%, sepsis 0.14%, haemorrhage 0.79%, and surgical site infection 1.25% [30]. For elective sigmoidectomy, a US study found similar frequencies for perioperative complications as seen with our data, with an overall in-hospital mortality 0.9%, sepsis 1.1%, pneumonia 1.5%, haemorrhage 2.1%, and surgical site infection 3.2% [31]. The most common postoperative complication after surgical haemorrhoidectomy is haemorrhage in roughly 2% of the cases [32].

Given the fact that private hospitals predominantly offer their services to patients with supplementary private insurance only, access to elective surgery for patients with supplementary private insurance were likely reduced during the pandemic. Therefore, our findings may be rather conservative than optimistic.

Given the differences in treatment allocation between patients with supplementary private insurance and basic health insurance only, one could argue that this also leads to differences in in-hospitals safety outcomes. It is reassuring that our data showed no significant differences in terms of in-hospital safety outcomes supporting the quality of care given to in-patients with non-emergency visceral surgical procedures, regardless of their respective insurance status.

### Limitations

The dataset used in this study was provided by the Federal Statistical Office (FSO) which applies different anonymization protocols based on the specific project and requested granularity of information. In our study, we focused on the granularity of clinical information, and thus, nationality was categorized by the FSO into a binary variable. It is worth noting that, unlike in the United States of America, race is usually not recorded or collection is even prohibited in European countries.

We acknowledge that this approach might potentially present a limitation. As a consequence, we sought additional publicly available data from the FSO reporting the percentage of individuals living with chronic health conditions in Switzerland stratified by nationality [33]. No substantial differences were identified between the nationalities represented, which is why we do not anticipate that the binarization of nationality into Swiss and non-Swiss categories introduces bias.

The rate of urgent or emergent surgeries is strongly influenced by the underlying disease. To maintain a homogenous sample, we specifically selected highly elective procedures only being only rarely performed as emergent surgeries. By excluding these procedures, we aimed to minimize potential biases.

However, we acknowledge that some of the procedures could still be performed in an emergency setting which might inflate our estimate. Despite this possibility, we believe in the importance of highlighting potential imbalances in health care access and any signal should be considered a warning sign for policymakers.

Given the limitations of billing data, we were only able to adjust for patient characteristics relevant to general statistics and reimbursement but were lacking further characteristics such as education, housing area, household income, laboratory parameters, hospital ownership, and physician seniority. Even though we re-evaluated and adapted the codes for every investigated procedure over the study years, misclassifications due to coding issues in hospitals cannot be ruled out completely. In addition, the database used contains information on in-patient procedures only, which is why we put our focus on procedures primarily performed in an in-patient setting to reduce selection bias. Furthermore, we have analysed a retrospective dataset which precludes any inferences on causality.

As royalties are only applicable to the in-patient setting, a strong financial incentive is generated. However, no public database exists on outpatient treatments and the precise proportion of supplementary private insurance plans is also not known [6]. Even if there might be some selection bias in our model, patients with supplementary private insurance should receive fewer procedures, since evidence indicates that they are on average healthier than people with basic insurance only [3]. Although, this might make our estimates liberal, it is concerning that sicker people who should preferentially be treated as in-patients could not get optimal care. Additionally, we made assumptions on person-time by aggregating it across age, sex, and admission year from publicly available data, potentially deviating from the true estimates.

## Conclusion

In this observational cohort study, we found an association between the presence of a supplementary private insurance and a higher rate of visceral surgical procedures compared to patients with basic insurance only. As this association seems to be independent of medical comorbidities, a heightened health-conscious behaviour by people with supplementary private insurance besides financial incentives could be drivers. Ideally, health care systems should be solely guided by quality of care and not by reimbursement structures. Thus, our work aims to encourage national and international policy makers to remove solely financially driven reimbursements and instead focus on incentives for quality of care.

## Supplementary Information

Supplementary Material 1.

## Data Availability

Data was provided by FSO (Federal Statistical Office or Bundesamt fuer Statistik, Switzerland, Gesundheitsversorgungsstatistik 2012–2020). According to Swiss law, FSO data can only be provided by the FSO itself. Thus, researchers interested in this data need to send a request directly to the FSO.

## Abbreviations

ATE: Average Treatment Effect
ATT: Average Effect of Treatment on the Treated
BMI: Body Mass Index
CHE: Cholecystectomy
CHOP: Swiss Classification of Operations
CI: Confidence Interval
COPD: Chronic Obstructive Pulmonary Disease
EKNZ BASEC: Institutional Review Board of Northwestern Switzerland
ESCP: European Society of Coloproctology
FHR: Femoral Hernia Repair
FSO: Federal Statistical Office
FundoP: Fundoplication
GDP: Gross Domestic Product
HRE: Haemorrhoidectomy
ICD-10 GM: International Classification of Diseases, Version 10, German Modification
ICU: Intensive Care Unit
IHR: Inguinal Hernia Repair
IR: Incidence Rate
IRR: Incidence Rate Ratio
IPTW: Inverse Probability of Treatment Weighting
IPW: Inverse Probability Weighting
IQR: Interquartile Range
LOS: Length of Stay
MACE: Major Adverse Cardiovascular Events
NOGA: General Classification of Economic Activities
OECD: Organization for Economic Cooperation and Development
OSA: Obstructive Sleep Apnea
PS: Propensity Score
PSM: Propensity Score Matching
QoL: Quality of Life
RPX: Rectopexy
SigmoidE: Sigmoidectomy
SMD: Standardized Mean Difference
SSI: Surgical Site Infection
STROBE: Strengthening the Reporting of Observational Studies in Epidemiology
SwissDRG: Swiss Diagnosis Related Groups
UHR: Umbilical Hernia Repair
UTI: Urinary Tract Infection

## References

[1] OECD, Union E. Health at a Glance: Europe 2022; 2022. https://www.oecd-ilibrary.org/content/publication/507433b0-en.

[2] Biller-Andorno N, Zeltner T. Individual Responsibility and Community Solidarity-The Swiss Health Care System. N Engl J Med. 2015;373(23):2193–7. 10.1056/NEJMp1508256. 26630139

[3] Roth S, Zufferey J, Pellegrini S. Personen mit und ohne Spitalzusatzversicherung. Vergleich der soziodemografischen Merkmale, des Gesundheitszustands und der Inanspruchnahme von Versorgungsleistungen. Neuchâtel: Bundesamt für Statistik; 2021. p. 8.

[4] Engelberger K. Akutstationäre Spitaltarife im Zusatzversicherungsbereich Ein nationaler Tarif- und Kostenvergleich. Eidgenössisches Departement für Wirtschaft, Bildung und Forschung WBF, Preisüberwachung PUE; 2021:17. https://www.preisueberwacher.admin.ch. Accessed Apr 2022.

[5] Bolt L, Wertli MM, Haynes AG, et al. Variation in regional implantation patterns of cardiac implantable electronic device in Switzerland. PLoS One. 2022;17(2):e0262959. 10.1371/journal.pone.0262959. PMC8849475 35171922

[6] Struja T, Suter F, Rohrmann S, et al. Comparison of Cardiovascular Procedure Rates in Patients With Supplementary vs Basic Insurance in Switzerland. JAMA Netw Open. 2023;6(1):e2251965–e2251965. 10.1001/jamanetworkopen.2022.51965. PMC9860525 36662521

[7] Wertli MM, Schlapbach JM, Haynes AG, et al. Regional variation in hip and knee arthroplasty rates in Switzerland: a population-based small area analysis. PLoS One. 2020;15(9):e0238287. 10.1371/journal.pone.0238287. PMC7505431 32956363

[8] Busato A, Widmer M, Matter P. Variation in incidence of orthopaedic surgery between populations with basic or basic plus supplementary health insurance in Switzerland. Swiss Med Wkly. 2011;141:w13152. 10.4414/smw.2011.13152. 21293980

[9] Muheim LLS, Senn O, Fruh M, Reich O, Rosemann T, Neuner-Jehle SM. Inappropriate use of arthroscopic meniscal surgery in degenerative knee disease. Acta Orthop. 2017;88(5):550–5. 10.1080/17453674.2017.1344915. PMC5560220 28665174

[10] Mehra T, Moos RM, Seifert B, et al. Impact of structural and economic factors on hospitalization costs, inpatient mortality, and treatment type of traumatic hip fractures in Switzerland. Arch Osteoporos. 2017;12(1):7. 10.1007/s11657-016-0302-3. 28013447

[11] (FSO) SFSO. Swiss Classification of Operations (CHOP). https://www.bfs.admin.ch/asset/de/18304268. Accessed 11 Mar 2023.

[12] ICD10GM. International Classification of Diseases, Version 10 (ICD-10 GM). Federal Institute for Drugs and Medical Devices (BfArM). https://www.icd-code.de. Accessed 11 Mar 2023.

[13] von Elm E, Altman DG, Egger M, et al. The Strengthening the Reporting of Observational Studies in Epidemiology (STROBE) statement: guidelines for reporting observational studies. J Clin Epidemiol. 2008;61(4):344–9. 10.1016/j.jclinepi.2007.11.008. 18313558

[14] Austin PC. Using the Standardized Difference to Compare the Prevalence of a Binary Variable Between Two Groups in Observational Research. Commun Stat Simul Comput. 2009;38(6):1228–34. 10.1080/03610910902859574.

[15] Office FS. Permanent resident population by age, canton, district and commune, 2010–2020. Federal Statistical Office. https://www.bfs.admin.ch/bfs/en/home/statistics/population/effectif-change/population.assetdetail.18344320.html. Accessed Mar 2023.

[16] Bruno Trezzinia BM. Erste Erfahrungen mit den Listen «ambulant vor stationär». Schweizerische Ärztezeitung: FMH; 2019.

[17] Allan V, Ramagopalan SV, Mardekian J, et al. Propensity score matching and inverse probability of treatment weighting to address confounding by indication in comparative effectiveness research of oral anticoagulants. J Comp Eff Res. 2020;9(9):603–14. 10.2217/cer-2020-0013. 32186922

[18] Matschinger H, Heider D, König HH. A Comparison of Matching and Weighting Methods for Causal Inference Based on Routine Health Insurance Data, or: What to do If an RCT is Impossible. Ein Vergleich von „matching“ und „weighting“-Verfahren zur Kausalanalyse mit Routinedaten von Krankenversicherungen, oder: Was tun wenn ein RCT nicht möglich ist. Gesundheitswesen. 2020;82(S 02):S139–50. 10.1055/a-1009-6634. 32066197

[19] Austin PC. Differences in target estimands between different propensity score-based weights. Pharmacoepidemiol Drug Saf. 2023;32(10):1103–12. 10.1002/pds.5639. 37208837

[20] Austin PC. An Introduction to Propensity Score Methods for Reducing the Effects of Confounding in Observational Studies. Multivariate Behav Res. 2011;46(3):399–424. 10.1080/00273171.2011.568786. PMC3144483 21818162

[21] Vuong KT, Guglielmetti LC, Albert TG, Brillat Arce W, Staerkle RF, Vuille-dit-Bille RN. Outpatient before inpatient—the good, the bad and the ugly. Eur Surg. 2021;53(1):11–5. 10.1007/s10353-020-00656-x.

[22] OECD. OECD Health Statistics. OECD. https://stats.oecd.org/Index.aspx?ThemeTreeId=9. Accessed 11 Mar 2023.

[23] Federal Office of Public Health S. Faktenblatt: Regelung "ambulant vor stationär". https://www.bag.admin.ch/dam/bag/de/dokumente/kuv-leistungen/Laufende%20Revisionsprojekte/ambulant-vor-stationaer/faktenblatt-ambulant-vor-stationaer-version-29042019.pdf.download.pdf/Faktenblatt%20zu%20Ambulant%20vor%20Station%C3%A4r,%20Version%20vom%2029.04.2019.pdf. Accessed 16 Apr 2023.

[24] Enodien B, Taha-Mehlitz S, Bachmann M, et al. Analysis of Factors Relevant to Revenue Enhancement in Hernia Interventions (SwissDRG G09). Healthcare (Basel). 2021;9(7) . 10.3390/healthcare9070862. PMC8306973 34356240

[25] Brüngger B, Bähler C, Schwenkglenks M, et al. Surgical procedures in inpatient versus outpatient settings and its potential impact on follow-up costs. Health Policy. 2021;125(10):1351–8. 10.1016/j.healthpol.2021.07.006. 34348846

[26] Ziga M, Burla L, Imhof A, Gürtler T, Weber M. Inguinal hernia treatment in Switzerland: inpatient vs. outpatient setting - HerStAmb Study (prospective observational two-centre comparative study). Langenbecks Arch Surg. 2023;408(1):14. 10.1007/s00423-023-02766-y. 36622458

[27] Janjua H, Cousin-Peterson E, Barry TM, Kuo MC, Baker MS, Kuo PC. Robotic Approach to Outpatient Inguinal Hernia Repair. J Am Coll Surg. 2020;231(1):61–72. 10.1016/j.jamcollsurg.2020.04.031. 32380165

[28] Schultz JK, Azhar N, Binda GA, et al. European Society of Coloproctology: guidelines for the management of diverticular disease of the colon. Colorectal Dis. 2020;22(S2):5–28. 10.1111/codi.15140. 32638537

[29] Baum P, Diers J, Lichthardt S, et al. Mortality and complications following visceral surgery. Dtsch Arztebl International. 2019;116(44):739–46. 10.3238/arztebl.2019.0739 PMC6912125 31774053

[30] Pucher PH, Brunt LM, Davies N, et al. Outcome trends and safety measures after 30 years of laparoscopic cholecystectomy: a systematic review and pooled data analysis. Surg Endosc. 2018;32(5):2175–83. 10.1007/s00464-017-5974-2. PMC5897463 29556977

[31] Ilyas MI, Zangbar B, Nfonsam VN, et al. Are there differences in outcome after elective sigmoidectomy for diverticular disease and for cancer? A national inpatient study. Colorectal Dis. 2017;19(3):260–5. 10.1111/codi.13461. 27422847

[32] Davis BR, Lee-Kong SA, Migaly J, Feingold DL, Steele SR. The American Society of Colon and Rectal Surgeons Clinical Practice Guidelines for the Management of Hemorrhoids. Dis Colon Rectum. 2018;61(3):284–92. 10.1097/dcr.0000000000001030. 29420423

[33] Federal Statistical Office. Population in Switzerland. https://www.bfs.admin.ch/bfs/en/home/statistics/population/effectif-change.html. Accessed 08.02.2025.

