# Supplementary material for: Association between health care insurance type and rates of visceral surgical procedures in Switzerland. A population-based weighted retrospective cohort study
Source: BMC Public Health. 2026 Jul 3;26:2840. doi: 10.1186/s12889-026-28164-9 (PMC13644274; doi:10.1186/s12889-026-28164-9)
Supplement: Supplementary file 1 [file 12889_2026_28164_MOESM1_ESM.docx]

**Supplementary Material**

**Association between health care insurance type and rates of visceral surgical procedures in Switzerland**

***A population-based weighted retrospective cohort study***

Kris Rafaisz^1, 2^, MD; Flurina Suter^3^, MSc; Sabine Rohrmann^3^, PhD, MPH; Beat Mueller^2,4^, MD; Philipp Schuetz^2,4^, MD, MPH; Christian Andreas Nebiker^4,5^, MD; Alexander Kutz^2,†^, MD, MSc, MPH; Tristan Struja^2,6,†^, MD, MSc, MPH

^†^ Shared senior authors

[kris.rafaisz@unibas.c](mailto:kris.rafaisz@unibas.c)h; [flurina.suter2@uzh.ch](mailto:flurina.suter2@uzh.ch); [sabine.rohrmann@uzh.ch](mailto:sabine.rohrmann@uzh.ch); [happy.mueller@unibas.ch](mailto:happy.mueller@unibas.ch); [schuetzph@gmail.com](mailto:schuetzph@gmail.com); [christian.nebiker@ksa.ch](mailto:christian.nebiker@ksa.ch); [kutz.alexander@gmail.com](mailto:kutz.alexander@gmail.com); [tristan.struja@gmail.com](mailto:tristan.struja@gmail.com)

^1^ Department of Internal Medicine, University Hospital Zurich (USZ), University of Zurich (UZH), Zurich, Switzerland

^2^ Medical University Clinic, Kantonsspital Aarau (KSA), Aarau, Switzerland

^3^ Division of Chronic Disease Epidemiology, Epidemiology, Biostatistics and Prevention Institute (EBPI), University of Zurich (UZH), Zurich, Switzerland

^4^ Medical Faculty of the University of Basel, Basel, Switzerland

^5^ Division of Visceral Surgery, Surgery, Kantonsspital Aarau (KSA), Aarau, Switzerland

^6^ Massachusetts Institute of Technology, Institute for Medical Engineering and Science, Cambridge, Massachusetts, USA

VERSION: June 07, 2026

KEYWORDS: health care, insurance, visceral surgery, procedure, quality of care

SUPPLEMENTARY MATERIAL: 5 tables, 6 figures

**Corresponding author and person to whom reprint requests should be addressed:**

Kris Rafaisz, MD

ORCID 0009-0007-9533-209X

Medical University Clinic, Kantonsspital Aarau (KSA)

Tellstrasse, 5001 Aarau, Switzerland

E-Mail: [kris.rafaisz@unibas.c](mailto:kris.rafaisz@unibas.c)h

**Supplemental Online Content**

eFigure 1: Diagram of the Swiss health care insurance and reimbursement system

eTable 1: Parameters included in logistic regression model to calculate propensity scores

eTable 2: Baseline characteristics in patients with main visceral surgical procedures

eFigure 2: Density of propensity scores before matching according to health insurance status

eFigure 3: Density of propensity scores after matching according to health insurance status

eFigure 4: Incidence rates of main and any visceral surgical procedures over the study years, according to health insurance status

eTable 3: CHOP codes of visceral surgical procedures over the study years

eFigure 5: Boxplot of logarithmic incidence rates for main visceral surgical procedures (panel a) and for any visceral surgical procedures (panel b) over deciles of age in years, stratified by health insurance status

eTable 4: Incidence rates, incidence rate differences, and additional annual treatments for main visceral surgical procedures, stratified by type of analysis

eFigure 6: Forest plot of incidence rate ratios for any (main and side) visceral surgical procedures, stratified by type of analysis

eTable 5: Incidence rates, incidence rate difference, and additional annual treatments for any (main and side) visceral surgical procedures

**eFigure 1: Diagram of the Swiss health care insurance and reimbursement system**

**eTable 1: Parameters included in logistic regression model to calculate propensity scores**

| **Outcome** | **Binary variables** | | **Categorical variables** | **Continuous variables** | **Interactions** |
| --- | --- | --- | --- | --- | --- |
| Insurance class:  Basic insurance as reference | Non-Swiss nationality | Atrial fibrillation | Hospital type/ size | Age | Age # Elixhauser |
|  | Sex | Peripheral artery disease | Anonymous hospital identifier | Year | Hospital type # Year |
|  | Living not at home | Cerebrovascular disease |  | Length of stay | Length of stay # Elixhauser |
|  | Type of clinic | Chronic kidney disease |  | Elixhauser comorbidity score |  |
|  | Obesity | End stage kidney disease |  |  |  |
|  | Dyslipidaemia | Chronic obstructive pulmonary disease |  |  |  |
|  | Obstructive sleep apnoea | Solid malignancy |  |  |  |
|  | Diabetes mellitus | Hematologic malignancy |  |  |  |
|  | Hypertension | Alcohol abuse |  |  |  |
|  | Coronary artery disease |  |  |  |  |

Legend: All 28 covariates were included in the PS model. We excluded 2% of the most extreme PS for the IPW analysis. For the 1:1 PS-matching, we used the nearest neighbour method with a maximum calliper of 0.01 on the PS scale. A standardized mean difference less than 0.10 was considered adequate for covariate balance after IPW and PS-matching. The variables available in the complete dataset are accessible on the FSO’s <https://www.bfs.admin.ch/bfs/de/home/statistiken/gesundheit/erhebungen/ms.assetdetail.12167417.html>.

**eTable 2: Baseline characteristics in patients with main visceral surgical procedures only**

|  | **Total**  **N=248,522** | **Basic insurance**  **N=181,238** | **Supplementary private insurance**  **N=67,284** |
| --- | --- | --- | --- |
| Age, years (median, IQR) | 58 (46–69) | 55 (43–67) | 64 (53–72) |
| Non-Swiss nationality, n (%) | 45,995 (18.5%) | 39,880 (22.0%) | 6,115 (9.1%) |
| Male sex, n (%) | 162,796 (65.5%) | 118,126 (65.2%) | 44,670 (66.4%) |
| Elixhauser comorbidity score (median, IQR) | 0.00 (0.00–1.00) | 0.00 (0.00–1.00) | 0.00 (0.00–1.00) |
| Charlson index (median, IQR) | 0.00 (0.00-0.00) | 0.00 (0.00-0.00) | 0.00 (0.00-0.00) |
| Living not at home, n (%) | 1,334 (0.5%) | 1,196 (0.7%) | 138 (0.2%) |
| *Hospital type, n (%)* |  |  |  |
| University | 11,670 (4.7%) | 10,269 (5.7%) | 1,401 (2.1%) |
| Tertiary | 149,157 (60.0%) | 114,107 (63.0%) | 35,050 (52.1%) |
| Secondary, large | 38,096 (15.3%) | 25,103 (13.9%) | 12,993 (19.3%) |
| Secondary, medium | 42,140 (17.0%) | 27,173 (15.0%) | 14,967 (22.2%) |
| Secondary, small | 7,459 (3.0%) | 4,586 (2.5%) | 2,873 (4.3%) |
| *Year, n (%)* |  |  |  |
| 2012 | 27,991 (11.3%) | 20,001 (11.0%) | 7,990 (11.9%) |
| 2013 | 28,732 (11.6%) | 20,736 (11.4%) | 7,996 (11.9%) |
| 2014 | 29,534 (11.9%) | 21,408 (11.8%) | 8,126 (12.1%) |
| 2015 | 29,443 (11.8%) | 21,488 (11.9%) | 7,955 (11.8%) |
| 2016 | 29,899 (12.0%) | 21,964 (12.1%) | 7,935 (11.8%) |
| 2017 | 29,832 (12.0%) | 22,065 (12.2%) | 7,767 (11.5%) |
| 2018 | 28,008 (11.3%) | 20,605 (11.4%) | 7,403 (11.0%) |
| 2019 | 23,635 (9.5%) | 17,221 (9.5%) | 6,414 (9.5%) |
| 2020 | 21,448 (8.6%) | 15,750 (8.7%) | 5,698 (8.5%) |
| Length of stay, days (median, IQR) | 2 (2–3) | 2 (2–3) | 2 (1–3) |
| Obesity, n (%) |  |  |  |
| BMI <30.0 kg/m^2^ | 244,565 (98.4%) | 177,920 (98.2%) | 66,645 (99.1%) |
| BMI <30.0–34.9 kg/m^2^ | 1,894 (0.8%) | 1,526 (0.8%) | 368 (0.5%) |
| BMI <35.0–39.9 kg/m^2^ | 1,204 (0.5%) | 1,012 (0.6%) | 192 (0.3%) |
| BMI ≥40.0 kg/m^2^ | 859 (0.3%) | 780 (0.4%) | 79 (0.1%) |
| *Comorbidities, n (%)* |  |  |  |
| Dyslipidaemia | 17,132 (6.9%) | 11,453 (6.3%) | 5,679 (8.4%) |
| OSA | 3,184 (1.3%) | 2,406 (1.3%) | 778 (1.2%) |
| Diabetes mellitus | 13,481 (5.4%) | 10,478 (5.8%) | 3,003 (4.5%) |
| Hypertension | 56,050 (22.6%) | 39,189 (21.6%) | 16,861 (25.1%) |
| Coronary artery disease | 13,059 (5.3%) | 8,801 (4.9%) | 4,258 (6.3%) |
| Atrial fibrillation | 7,860 (3.2%) | 5,271 (2.9%) | 2,589 (3.8%) |
| Peripheral artery disease | 1,677 (0.7%) | 1,235 (0.7%) | 442 (0.7%) |
| Cerebrovascular disease | 1,000 (0.4%) | 697 (0.4%) | 303 (0.5%) |
| Chronic kidney disease | 3,882 (1.6%) | 2,835 (1.6%) | 1,047 (1.6%) |
| End-stage kidney disease | 318 (0.1%) | 238 (0.1%) | 80 (0.1%) |
| COPD | 3,512 (1.4%) | 2,722 (1.5%) | 790 (1.2%) |
| Solid malignancy | 4,385 (1.8%) | 3,157 (1.7%) | 1,228 (1.8%) |
| Hematologic malignancy | 407 (0.2%) | 276 (0.2%) | 131 (0.2%) |
| Alcohol abuse | 1,029 (0.4%) | 903 (0.5%) | 126 (0.2%) |
| Liver cirrhosis | 664 (0.3%) | 531 (0.3%) | 133 (0.2%) |
| Congestive heart disease | 952 (0.4%) | 704 (0.4%) | 248 (0.4%) |
| *Main procedures, n (%)* |  |  |  |
| Cholecystectomy | 67,140 (27.0%) | 51,602 (28.5%) | 15,538 (23.1%) |
| Fundoplication | 3,996 (1.6%) | 2,802 (1.5%) | 1,194 (1.8%) |
| Sigmoidectomy | 13,792 (5.5%) | 9,434 (5.2%) | 4,358 (6.5%) |
| Rectopexy | 3,279 (1.3%) | 2,144 (1.2%) | 1,135 (1.7%) |
| Haemorrhoidectomy | 18,197 (7.3%) | 12,738 (7.0%) | 5,459 (8.1%) |
| Inguinal hernia | 116,686 (47.0%) | 82,855 (45.7%) | 33,831 (50.3%) |
| Femoral hernia | 3,064 (1.2%) | 2,224 (1.2%) | 840 (1.2%) |
| Umbilical hernia | 22,368 (9.0%) | 17,439 (9.6%) | 4,929 (7.3%) |
| Any hernia procedure | 142,118 (57.2%) | 102,518 (56.6%) | 39,600 (58.9%) |
| Overall main procedures | 248,522 (100.0%) | 181,238 (100.0%) | 67,284 (100.0%) |

Abbr.: BMI, body mass index; OSA, obstructive sleep apnoea; COPD, chronic obstructive pulmonary disease.

**eFigure 2: Density of propensity scores before matching according to health insurance status**

**eFigure 3: Density of propensity scores after matching according to health insurance status**

**eFigure 4:** **Incidence rates of main and any visceral surgical procedures over the study years, according to health insurance status**

**a and b main and any procedure cholecystectomy (CHE), respectively**

**c and d main and any procedure fundoplication (FundoP), respectively**

| **a** |  | **b** |  |
| --- | --- | --- | --- |
| **c** |  | **d** |  |

**e and f main and any procedure sigmoidectomy (SigmoidE), respectively**

**g and h main and any procedure rectopexy (RPX), respectively**

| **e** |  | **f** |  |
| --- | --- | --- | --- |
| **g** |  | **h** |  |

**i and j main and any procedure haemorrhoidectomy (HRE), respectively**

**k and l main and any procedure inguinal hernia repair (IHR), respectively**

| **i** |  | **j** |  |
| --- | --- | --- | --- |
| **k** |  | **l** |  |

**m and n main and any procedure femoral hernia repair (FHR), respectively**

**o and p main and any procedure umbilical hernia repair (UHR), respectively**

| **m** |  | **n** |  |
| --- | --- | --- | --- |
| **o** |  | **p** |  |

**q and r main and any procedure all hernia repairs (AHR), respectively**

| **q** |  | **r** |  |
| --- | --- | --- | --- |

Abbr.: 99% CI, confidence interval; CHE, cholecystectomy; FundoP, fundoplication; SigmoidE, sigmoidectomy; RPX, rectopexy; HRE, haemorrhoidectomy; IHR, inguinal hernia repair; FHR, femoral hernia repair; UHR, umbilical hernia repair; AHR, all hernia repairs.

**eTable 3:** **CHOP codes of visceral surgical procedures over the study years**

| **CHOP Code** | **Description** | **Procedure** | **Year 2012** | **Year 2013** | **Year 2014** | **Year 2015** | **Year 2016** | **Year 2017** | **Year 2018** | **Year 2019** | **Year 2020** |
| --- | --- | --- | --- | --- | --- | --- | --- | --- | --- | --- | --- |
| 51.22 | Cholecystectomy, excl. LSC cholecystectomy | CHE | X | X | X | X | X | X | X | X | X |
| 51.23 | Laparoscopic Cholecystectomy | CHE | X | X | X | X | X | X | X | X | X |
| 51.24 | Partial Laparoscopic Cholecystectomy | CHE | X | X | X | X | X | X | X | X | X |
| 44.66 | Other Procedures for Reconstruction of Esophagogastric Sphincter Function: Fundoplication, Nissen Fundoplication, Gastric Cardia Plastic, Reconstruction of Cardioesophageal Angle | FundoP | X | X | X | X | X | X | X | X | X |
| 44.67 | Laparoscopic Procedures for Reconstruction of Esophagogastric Sphincter Function: Nissen Fundoplication, Reconstruction of Cardioesophageal Angle | FundoP | X | X | X | X | X | X | X | X | X |
| 45.76 | Sigmoidectomy* | SigmoidE | X | X | X | X | X | X | X | X | X |
| 45.75 | Left Hemicolectomy* | SigmoidE | X | X | X | X | X | X | X | X | X |
| 4575.2 | Left Hemicolectomy with Sigmoidectomy* | SigmoidE |  |  |  |  |  | X | X | X | X |
| 45.79.21 | Extended Right and Left Hemicolectomy* | SigmoidE | X | X | X | X |  |  |  |  |  |
| 45.79.25 | Extended Left Hemicolectomy, Open Surgery* | SigmoidE |  |  |  |  | X |  |  |  |  |
| 45.79.26 | Extended Left Hemicolectomy, Laparoscopic* | SigmoidE |  |  |  |  | X |  |  |  |  |
| 48.75 | Abdominal Rectopexy | RPX | X | X | X | X | X | X | X | X | X |
| 48.76 | Other Rectopexy | RPX | X | X | X | X | X | X | X | X | X |
| 49.46 | Excision Haemorrhoids | HRE | X | X | X | X | X | X | X | X | X |
| 53.0 | (Unilateral) Inguinal Hernia | IHR | X | X | X | X | X | X | X | X | X |
| 53.1 | Bilateral Inguinal Hernia | IHR | X | X | X | X | X |  |  |  |  |
| 53.2 | (Unilateral) Femoral Hernia | FHR | X | X | X | X | X | X | X | X | X |
| 53.3 | Bilateral Femoral Hernia | FHR | X | X | X | X | X |  |  |  |  |
| 53.4 | Umbilical Hernia | UHR | X | X | X | X | X | X | X | X | X |

Legend: CHOP codes marked by an “X” are the ones used in the individual year as CHOP codes are changed annually.

Abbr.: CHE, cholecystectomy; FundoP, fundoplication; SigmoidE, sigmoidectomy; RPX, rectopexy; HRE, haemorrhoidectomy; IHR, inguinal hernia repair; FHR, femoral hernia repair; UHR, umbilical hernia repair; AHR, all hernia repairs.

*CHOP codes used in conjunction with ICD-Codes for colonic diverticulosis (K57.30) and colonic diverticulitis (K57.32), both excluding perforation, abscess, or bleeding.

**eFigure 5: Boxplot of logarithmic incidence rates for main visceral surgical procedures (panel a) and for any visceral surgical procedures (panel b) over deciles of age in years, stratified by health insurance status**

| **a** |  | **b** |  |
| --- | --- | --- | --- |

Legend: IR, incidence rate; horizontal bar denotes median, and edges of rectangles the 25^th^ and 75^th^ percentiles, whiskers extend to include all data points within 1.5 times the interquartile range, circles denote outside values.

**eTable 4: Incidence rates, incidence rate differences, and additional annual treatments for main visceral surgical procedures, stratified by type of analysis**

| **Outcome** | **Model ^†^** | **IR** ^¶^  **Basic insurance** | **IR** ^¶^  **Private insurance** | **IR**  **Difference ^*^** | **99%**  **CI** | **Additional annual procedures ^‡^** |
| --- | --- | --- | --- | --- | --- | --- |
| Cholecystectomy main | Crude | 130.55 | 144.32 | 13.77 | 13.58 to 13.96 | 165 |
|  | IPW | 134.23 | 142.20 | 7.97 | 7.76 to 8.17 |  |
|  | IPW LOS ≤ 7d | 131.67 | 139.76 | 8.10 | 7.87 to 8.33 |  |
|  | PS Matching | 140.14 | 142.21 | 2.06 | 1.80 to 2.33 |  |
|  | CEM | 141.98 | 144.40 | 2.42 | 2.16 to 2.68 |  |
| Fundoplication main | Crude | 8.49 | 10.06 | 1.57 | 1.54 to 1.60 | 17 |
|  | IPW | 8.90 | 9.71 | 0.81 | 0.78 to 0.85 |  |
|  | IPW LOS ≤ 7d | 8.60 | 9.38 | 0.78 | 0.74 to 0.81 |  |
|  | PS Matching | 9.56 | 9.76 | 0.20 | 0.16 to 0.25 |  |
|  | CEM | 9.88 | 10.14 | 0.26 | 0.22 to 0.31 |  |
| Sigmoidectomy main | Crude | 32.97 | 39.54 | 6.57 | 6.48 to 6.65 | 73 |
|  | IPW | 34.75 | 38.26 | 3.52 | 3.42 to 3.61 |  |
|  | IPW LOS ≤ 7d | 33.43 | 36.92 | 3.49 | 3.39 to 3.60 |  |
|  | PS Matching | 37.58 | 38.38 | 0.80 | 0.68 to 0.92 |  |
|  | CEM | 39.30 | 39.97 | 0.67 | 0.56 to 0.79 |  |
| Rectopexy  main | Crude | 6.58 | 8.55 | 1.97 | 1.92 to 2.01 | 8 |
|  | IPW | 7.31 | 7.69 | 0.37 | 0.33 to 0.42 |  |
|  | IPW LOS ≤ 7d | 6.63 | 6.97 | 0.34 | 0.29 to 0.38 |  |
|  | PS Matching | 8.24 | 7.98 | -0.26 | -0.32 to -0.19 |  |
|  | CEM | 8.14 | 8.44 | 0.31 | 0.24 to 0.37 |  |
| Haemorrhoidectomy main | Crude | 34.11 | 34.96 | 0.86 | 0.79 to 0.93 | 25 |
|  | IPW | 34.35 | 35.56 | 1.21 | 1.13 to 1.29 |  |
|  | IPW LOS ≤ 7d | 34.23 | 35.60 | 1.37 | 1.28 to 1.45 |  |
|  | PS Matching | 34.84 | 35.15 | 0.31 | 0.21 to 0.42 |  |
|  | CEM | 35.11 | 34.94 | -0.17 | -0.27 to -0.07 |  |
| Inguinal hernia repair main | Crude | 295.46 | 331.64 | 36.18 | 34.96 to 37.40 | 479 |
|  | IPW | 306.30 | 329.46 | 23.16 | 21.89 to 24.42 |  |
|  | IPW LOS ≤ 7d | 298.99 | 323.44 | 24.45 | 23.07 to 25.83 |  |
|  | PS Matching | 323.30 | 327.53 | 4.22 | 2.51 to 5.94 |  |
|  | CEM | 346.89 | 339.95 | -6.94 | -8.68 to -5.20 |  |
| Femoral hernia repair main | Crude | 5.82 | 6.83 | 1.01 | 0.99 to 1.03 | 10 |
|  | IPW | 6.10 | 6.56 | 0.46 | 0.44 to 0.48 |  |
|  | IPW LOS ≤ 7d | 5.89 | 6.34 | 0.45 | 0.43 to 0.47 |  |
|  | PS Matching | 6.54 | 6.61 | 0.08 | 0.05 to 0.10 |  |
|  | CEM | 6.60 | 6.78 | 0.18 | 0.15 to 0.21 |  |
| Umbilical hernia repairs main | Crude | 44.15 | 43.14 | -1.01 | -1.13 to -0.88 | 23 |
|  | IPW | 43.77 | 44.86 | 1.09 | 0.96 to 1.22 |  |
|  | IPW LOS ≤ 7d | 44.30 | 45.65 | 1.35 | 1.21 to 1.50 |  |
|  | PS Matching | 43.49 | 43.84 | 0.35 | 0.17 to 0.52 |  |
|  | CEM | 45.33 | 43.88 | -1.45 | -1.62 to -1.27 |  |
| All hernia repairs main | Crude | 345.32 | 381.32 | 36.00 | 34.70 to 37.30 | 511 |
|  | IPW | 355.98 | 380.69 | 24.71 | 23.36 to 26.07 |  |
|  | IPW LOS ≤ 7d | 349.02 | 375.28 | 26.26 | 24.78 to 27.74 |  |
|  | PS Matching | 373.05 | 377.77 | 4.72 | 2.89 to 6.56 |  |
|  | CEM | 398.59 | 390.36 | -8.23 | -10.08 to -6.37 |  |
| Overall main procedures | Crude | 557.85 | 617.68 | 59.83 | 58.52 to 61.15 | 793 |
|  | IPW | 575.02 | 613.40 | 38.39 | 37.00 to 39.78 |  |
|  | IPW LOS ≤ 7d | 563.27 | 603.42 | 40.15 | 38.62 to 41.68 |  |
|  | PS Matching | 602.38 | 610.42 | 8.04 | 6.19 to 9.89 |  |
|  | CEM | 632.11 | 627.11 | -5.00 | -6.85 to -3.15 |  |

**^†^** All models were fit with a negative binomial regression with the dispersion parameter set at the mean count of the outcome. The crude model was fit just with the outcome and a binary variable for insurance class.

^¶^ Incidence rates are per 100,000 person-years.

^*^ Inconsistencies are due to rounding. Incidence difference is per 100,000 person-years.

**^‡^** Additional procedures per year in Switzerland: IRD/100,000*6.88 mio*0.3; 6.88 mio, population ≥18 years old in Switzerland on average from 2012 to 2020; 0.3, fraction of population with supplementary private insurance. Values are based on IPW model.

Abbr.: 99% CI, confidence interval; IPW, inverse probability weighting; IR, incidence rates; LOS, length of stay; PS, propensity score, CEM, coarsened exact matching.

**eFigure 6:** **Forest plot of incidence rate ratios for any (main and side) visceral surgical procedures, stratified by type of analysis**

Note: Inconsistencies with borderline confidence intervals are due to rounding. P-value <0.01 denotes a significant result at the 1% alpha level.

Legend: CI, confidence interval; crude model, model between outcome and insurance class without adjustment; IPW, inverse probability weighted model; LOS ≤ 7, IPW model with LOS range of 1–7 days instead of 1–28 days; PS, propensity score matching model.

**eTable 5: Incidence rates, incidence rate difference, and additional annual treatments for any (main and side) visceral surgical procedures**

| **Outcome** | **Model ^†^** | **IR** ^¶^  **Basic insurance** | **IR** ^¶^  **Private insurance** | **IR**  **Difference ^*^** | **99%**  **CI** | **Additional annual procedures ^‡^** |
| --- | --- | --- | --- | --- | --- | --- |
| Cholecystectomy any | Crude | 160.68 | 178.29 | 17.61 | 17.37 to 17.84 | 214 |
|  | IPW | 165.35 | 175.70 | 10.35 | 10.10 to 10.60 |  |
|  | IPW LOS ≤ 7d | 162.02 | 172.55 | 10.53 | 10.25 to 10.81 |  |
|  | PS Matching | 172.91 | 175.68 | 2.77 | 2.45 to 3.09 |  |
|  | CEM | 175.76 | 178.49 | 2.73 | 2.42 to 3.04 |  |
| Fundoplication  any | Crude | 12.66 | 14.92 | 2.26 | 2.22 to 2.30 | 25 |
|  | IPW | 13.26 | 14.43 | 1.18 | 1.13 to 1.22 |  |
|  | IPW LOS ≤ 7d | 12.84 | 13.97 | 1.13 | 1.08 to 1.18 |  |
|  | PS Matching | 14.21 | 14.50 | 0.29 | 0.23 to 0.35 |  |
|  | CEM | 14.65 | 15.03 | 0.38 | 0.32 to 0.44 |  |
| Sigmoidectomy  any | Crude | 36.64 | 44.23 | 7.59 | 7.50 to 7.68 | 82 |
|  | IPW | 38.72 | 42.66 | 3.94 | 3.84 to 4.04 |  |
|  | IPW LOS ≤ 7d | 37.12 | 41.03 | 3.91 | 3.80 to 4.02 |  |
|  | PS Matching | 42.00 | 42.86 | 0.86 | 0.73 to 0.99 |  |
|  | CEM | 43.88 | 44.64 | 0.76 | 0.63 to 0.88 |  |
| Rectopexy  any | Crude | 7.99 | 10.34 | 2.34 | 2.29 to 2.39 | 10 |
|  | IPW | 8.86 | 9.33 | 0.48 | 0.42 to 0.53 |  |
|  | IPW LOS ≤ 7d | 8.05 | 8.49 | 0.44 | 0.38 to 0.49 |  |
|  | PS Matching | 9.96 | 9.68 | -0.28 | -0.35 to -0.20 |  |
|  | CEM | 9.85 | 10.21 | 0.36 | 0.29 to 0.44 |  |
| Haemorrhoidectomy  any | Crude | 38.26 | 39.37 | 1.11 | 1.03 to 1.19 | 29 |
|  | IPW | 38.58 | 39.98 | 1.40 | 1.31 to 1.48 |  |
|  | IPW LOS ≤ 7d | 38.41 | 39.98 | 1.57 | 1.47 to 1.66 |  |
|  | PS Matching | 39.18 | 39.54 | 0.36 | 0.25 to 0.47 |  |
|  | CEM | 39.55 | 39.35 | -0.20 | -0.31 to -0.09 |  |
| Inguinal hernia repair any | Crude | 310.84 | 350.05 | 39.21 | 37.93 to 40.48 | 516 |
|  | IPW | 322.52 | 347.48 | 24.96 | 23.63 to 26,29 |  |
|  | IPW LOS ≤ 7d | 314.60 | 340.90 | 26.30 | 24.85 to 27.75 |  |
|  | PS Matching | 340.83 | 345.52 | 4.69 | 2.89 to 6.49 |  |
|  | CEM | 365.86 | 358.81 | -7.05 | -8.88 to -5.23 |  |
| Femoral hernia repair any | Crude | 11.03 | 12.92 | 1.89 | 1.86 to 1.92 | 19 |
|  | IPW | 11.55 | 12.45 | 0.90 | 0.87 to 0.93 |  |
|  | IPW LOS ≤ 7d | 11.16 | 12.05 | 0.89 | 0.85 to 0.92 |  |
|  | PS Matching | 12.36 | 12.50 | 0.14 | 0.10 to 0.18 |  |
|  | CEM | 12.73 | 12.92 | 0.19 | 0.15 to 0.23 |  |
| Umbilical hernia repairs any | Crude | 87.50 | 89.89 | 2.39 | 2.13 to 2.64 | 91 |
|  | IPW | 87.90 | 92.27 | 4.37 | 4.10 to 4.63 |  |
|  | IPW LOS ≤ 7d | 88.13 | 92.97 | 4.85 | 4.55 to 5.14 |  |
|  | PS Matching | 89.10 | 90.51 | 1.40 | 1.05 to 1.76 |  |
|  | CEM | 94.10 | 91.91 | -2.19 | -2.55 to -1.83 |  |
| All hernia repairs any | Crude | 381.75 | 422.99 | 41.25 | 39.85 to 42.65 | 584 |
|  | IPW | 393.76 | 422.05 | 28.29 | 26.83 to 29.76 |  |
|  | IPW LOS ≤ 7d | 385.92 | 415.88 | 29.97 | 28.36 to 31.57 |  |
|  | PS Matching | 413.06 | 418.86 | 5.80 | 3.82 to 7.78 |  |
|  | CEM | 441.28 | 432.95 | -8.33 | -10.33 to 6.32 |  |
| Overall any procedures | Crude | 623.15 | 692.37 | 69.22 | 67.78 to 70.65 | 913 |
|  | IPW | 642.79 | 686.98 | 44.19 | 42.68 to 45.71 |  |
|  | IPW LOS ≤ 7d | 629.19 | 675.28 | 46.09 | 44.42 to 47.76 |  |
|  | PS Matching | 674.15 | 683.85 | 9.71 | 7.69 to 11.72 |  |
|  | CEM | 707.22 | 702.61 | -4.61 | -6.62 to -2.59 |  |

**^†^** All models were fit with a negative binomial regression with the dispersion parameter set at the mean count of the outcome. The crude model was fit just with the outcome and a binary variable for insurance class.

^¶^ Incidence rates are per 100,000 person-years.

^*^ Inconsistencies are due to rounding. IR difference is per 100,000 person-years.

**^‡^** Additional procedures per year in Switzerland: IRD/100,000*6.88 mio*0.3; 6.88 mio, population ≥18 years old in Switzerland on average from 2012 to 2020; 0.3, fraction of population with supplementary private insurance. Values are based on IPW model.

Abbr.: 99% CI, confidence interval; IPW, inverse probability weighting; IR, incidence rates; LOS, length of stay; PS, propensity score, CEM, coarsened exact matching.
